# A Responsible Airtime Approach for True Time-Based Fairness in Multi-Rate WiFi Networks

**DOI:** 10.3390/s18113658

**Published:** 2018-10-28

**Authors:** Sang il Yu, Chang Yun Park

**Affiliations:** School of Computer Science and Engineering, Chung-Ang University, Seoul 06974, Korea; sangil_yu@cnlab.cse.cau.ac.kr

**Keywords:** airtime fairness, time-based fairness, performance anomaly, responsible airtime, fairness index, deficit round-robin, WiFi sensor networks, multi-rate wireless networks

## Abstract

Airtime fairness, or time-based fairness, has been well recognized as a method to solve WiFi performance anomalies and provide a balance between fairness and spectrum efficiency in multi-rate wireless networks. However, the definition of airtime is vague and simplistic. In this paper, it is demonstrated that current airtime fair scheduling results in unfairness in reality because overheads are neglected or unfairly counted. We introduce a notion of responsible airtime, which covers not only the data transmission time, but also all overheads, even a TCP ACK segment in TCP traffic. An approach based on responsible airtime can provide true time-based fairness, but responsible airtime is too complicated to directly handle. A practical method is thus introduced for evaluating responsible airtime fairness indirectly via throughput measurement. The key element, throughput fair share, of a node, is based on the baseline property in time-based fairness. For each node, an achieving ratio of actual throughput to the throughput fair share is determined, and a new fairness index considering deficiency as well as equity is applied. To validate the feasibility of responsible airtime fairness, we have developed a simple responsible airtime fair scheduler in access points for download traffic. Extensive simulation experiments are conducted in various network and traffic environments using the ns3 simulator. The results show that true time-based fairness is achievable in practice.

## 1. Introduction

Since the data rate capability and channel condition of nodes may differ from each other in a wireless network, a WiFi network operates at multiple data rates. It is well known that employing multiple data rates provides individual fairness; however, it causes WiFi performance anomalies [1], where the performance of a node using a higher data rate can be degraded to that of another node using a lower data rate. For the 802.11 wireless standard, increasingly faster standards continue to be released with backward compatibility. Moreover, various devices are often connected to an access point (AP), from a CCTV camera to a WiFi-enabled sensor. As a result, it is nowadays usual that a link is shared by devices at multiple data rates, ranging from a few Mbps to several hundred Mbps, and the performance is mostly determined by traffic at those slower nodes.

Most studies have addressed this performance anomaly by applying the idea of airtime fairness, or, more generally, time-based fairness. In CSMA/CA the chances of accessing the link are divided fairly among the nodes, but most of the link time is consumed by a lower data rate node. To solve the performance anomaly, the link times, rather than the chances, should be fairly divided. This is called airtime in the meaning of time to air signals to the link for transmission. An ideal example of true time-based fairness is the synchronous time-division multiple access (TDMA) in GSM mobile networks. Airtime fairness does not provide individual throughput fairness but rather provides fairness with respect to the system resources, i.e., link bandwidth. It additionally provides better spectral efficiency and, hence, higher overall throughput because the link is used a greater number of times at higher data rates. Airtime fairness has been recognized as equivalent to proportional fairness, which means that no better allocation exists in proportion to the rate capability, or speed ability, of nodes. 

However, practical experience with airtime fairness usually shows that results are biased against nodes with lower data rates: slower nodes have a lower throughput than expected and even unstable network connectivity. This study, first, figures out why most airtime fairness scheduling implementations are not fair in practice. The main reason is that airtime is treated in an incomplete way. Sending a data frame requires not only time to air the frame, but also overhead such as inter- frame spaces and ACK frames. Variable backoff delays are added, and even the time to transmit data from the receiver of the frame may be indirectly imposed. For example, a TCP ACK message, which should be sent by TCP on successful receiving data, is a derivative of the data and hence could be considered overhead, if it is not piggy-backed in time. It is obvious that faster nodes send more data in airtime fairness and more data accompanies more overheads. If a scheduler simply allocates an equal amount of time without correctly counting overheads, it results in unfairness in the real link time usage. In effect, a faster node uses more link time than a slower node.

True time-based fairness should concern not only the time of transmitting a data frame, but also the total time needed for the transmission of the frame. In this study, we introduce a new notion called responsible airtime for the latter, while the former is explicitly called pure airtime. There have been studies to extend airtime to cover overheads; for example, the concept of total airtime in [2] includes time cost from collisions. However, to our best knowledge, no attempt has been made to cover total responsible time including indirect cost such as TCP ACK. If fair scheduling is made based on responsible airtime, time-based fairness is achievable. However, unlike pure airtime, the exact responsible airtime of a frame is not easily calculable with its data length and data rate. It is also difficult to measure or analyze the actual responsible airtimes in a scheduling result, which are necessary to evaluate the time-based fairness of an implementation. This study approaches responsible airtime in an indirect and practical way. 

In perfect time-based fairness, each node uses the same amount of responsible airtime regardless of the data rate. We call this amount the responsible airtime fair share. In principle, this responsible airtime fair share is the basis of evaluating the fairness of a scheduling result. Throughput, not responsible time, is easily measurable. Therefore, we introduce throughput fair share, which is the notion of a fair share in throughput for responsible airtime fairness. The throughput fair share of a node is the throughput that the node achieves for its responsible airtime fair share. The responsible airtime fair share is the same for all the nodes; however, the throughput fair share may be different depending on the data rate of each node. In principle, the ratio of the actual responsible airtime to the responsible airtime fair share for each node is the basic element in the evaluation of our time-based fairness approach. In practice, it is indirectly derived from the ratio of the actual throughput to the throughput fair share of the node. 

The concept of throughput fair share has been mentioned in earlier studies as a baseline property [3], meaning that if airtime fairness is achieved in a multi-rate network, the throughput of a node is equal to the throughput of the node in the case in which all other nodes operate with the same data rate as the node. An example of perfect time-based fairness is the case where all nodes in a wireless network on the same channel condition including the data rate. In true time-based fairness the throughput of a node in this case should be kept the same, no matter how other nodes change their data rates. We believe that this baseline property is not only the consequence of airtime fairness, but it is also a sufficient and necessary condition for true time-based fairness. That is, whether the baseline property is satisfied can be used as a barometer for responsible airtime fairness. Our throughput fair share of a node corresponds to the throughput to be maintained in the baseline property.

The novelty of this study lies in that it separates the evaluation of fairness from analysis and allocation of airtime. Most existing studies calculate airtime by analyzing its components, and then fairness is implicitly ensured by allocating an equal amount of airtime to each node. In this approach, the correctness of the fairness evaluation depends upon the precision and completeness of the analysis. In other words, if the analysis is incomplete, the resulting fairness loses meaning. Our evaluation method is independent of the analysis and allocation method, and the fairness index can be used as an absolute measure.

A new fairness index is also proposed to evaluate the degree of time-based fairness for a given scheduling result. Most fairness indices address equity in performance metrics, and equity in actual responsible airtimes among nodes is important also in our study. However, in time-based fairness, where a node relinquishes equal performance to pursue system fairness, preserving each fair share could be critical. We add a notion of deficiency to equity to explicitly describe the extent of the shortage from the fair share that occurs at each node because equity does not distinguish surplus and shortage. 

Finally, we have developed a simple implementation of a responsible airtime fair scheduler at the AP. Our research goal is to validate the feasibility of responsible airtime fairness. Hence, we limit our development to a simple approximation of responsible airtime fair scheduling for download traffic. It keeps measuring samples of time for which transmission of a data frame is completed, from requesting a medium access to receiving the corresponding MAC ACK. This time sample contains most of the overheads including the backoff time as well as the transmission time of the data frame. An expected responsible time of a data frame for each destination node is calculated from the samples, and a scheduling algorithm that is basically similar to the deficit round-robin is applied. Accordingly, whenever a data frame to a node is transmitted, the expected responsible time to the node is counted as a deficit, and the next frame to that node can be scheduled after its deficit is cleared.

Our implementation scheme takes a system approach rather than an analytic approach. Instead of analyzing the components of costs one by one, the costs are estimated with a larger granularity, in an integrated manner. Using a larger granularity in the estimation process has the potential to avoid complications or imprecision arising from localized analysis. Moreover, a system approach gives a better chance of incorporating otherwise hidden indirect time costs. This approach is limited in precisely accounting for some overheads such as frame collisions. The implementation is independent of the physical layer. Thus, it is implementable purely in the queue management. It is applicable to any of the 802.11 physical standards or data rate adaptation mechanisms that are used. Through comprehensive simulations, it is shown that our simple implementation produces fairness results that are close to the ideal time-based fairness.

The remainder of this paper is organized as follows: Section 2 describes related work. Section 3 explains why existing airtime fairness scheduling is not fair in practice; in addition, the notion of responsible airtime is explained. An evaluation method through the throughput measurement is explained in Section 4. Section 5 presents a simple implementation of responsible airtime fair scheduling. The simulation experiments are described in Section 6. Finally, Section 7 concludes the study.

## 2. Related Work

Airtime fairness, or more generally, time-based fairness, in multi-rate wireless LAN has been studied primarily to solve performance anomalies [1]. Since performance anomalies are due to random access of the CSMA MAC, which pursues fairness in throughputs of individual nodes [4], solutions exist sacrificing this individual fairness. The other fairness criterion is fairness in the usage of system resources. Airtime fairness pursues fairness in the time for accessing the shared link, which is equivalent to fairness in bandwidth usage. There are variations in implementation, such as token-bucket regulator [4], airtime deficit round-robin scheduler [5], contention window controller [6], and 802.11e TXOP [7]. Nonetheless, they share the same goal of fair link use in time.

Some studies employ the notion of proportional fairness rather than airtime fairness. Since the performance anomaly is incurred while providing max-min fairness to individual nodes, the key for the solution is redefining the quality of fairness to enable a balance between fairness and spectrum efficiency. In multi-rate wireless LANs, proportional fairness basically means that the individual throughputs of each node are proportional to its data rate capability [6,7]. It was determined that proportional fairness is equivalent to airtime fairness in multi-rate wireless LANs [8,9]. Specifically, airtime scheduling provides proportional fairness, and a proportional fair allocation results in equal usage of airtime. Ref. [10] introduced a modified CSMA algorithm achieving proportional fairness, and compared the performances of various fairness criteria including time fairness. 

The baseline property was first mentioned in [3] as a result of airtime fair scheduling; however, it was not further explored. This study extends the property as a sufficient and necessary condition for airtime fairness. We employ it as a barometer to assess airtime fairness. Our notion of responsible airtime is conceptually simple but practically difficult to determine. From the baseline property, a proportional fair share in throughput of a node can be determined, and fairness is decided based on how closely each fair share is achieved in reality.

There have been many analytic studies for airtime or proportional fair allocation. For example, Reference [2,11] performed rigorous analysis to calculate airtime by taking into account most overheads including cost of collisions and developed an allocation scheme providing proportional fairness. Reference [12] developed an extensive analytic model for 802.11 DCF with general traffic loads and introduced a modified proportional fairness criterion for a practical allocation solution. Reference [13] addressed proportional fair allocation in MU-MIMO wireless LANs. It suggested that the notion of airtime should be subdivided into flow airtime and station airtime, because multiple flow transmissions can be made in parallel to multiple stations. Although overheads are not much concerned, its analysis showed that allocating equal airtime may not provide proportional fairness in a MU-MIMO environment.

In recent years, studies on airtime fairness in wireless networks have diverged. One approach is to extend its application to wireless networks other than a wireless LAN [14,15,16]. For example, Reference [14] addresses a time-based fairness approach in 5G networks. The other approach is to adequately develop it enough to be applicable to wireless routers in the real world. For example, Cisco implements an airtime fairness functionality in their commercial wireless routers [17]. The authors of [18] developed Linux kernel AP software with airtime fair scheduling and distributed it as open-source software. Reference [2] developed a light-weight implementation using only software, which is applicable to a commercial WiFi device. Reference [19] implemented a portable airtime allocation scheduler, which runs on any Linux-based WiFi device. Reference [20] developed a device enhancing the performance in an 802.11 infrastructure wireless LAN environment by alleviating performance anomalies including multi-rate anomaly. All implementations were done within a single box, without any modifications to the AP or the nodes. However, it does not address fairness as a primary concern; there is no fairness evaluation of the enhancement results.

Our study differs from existing ones in that it is more fundamental. That is, it addresses what airtime is exactly and how airtime fairness is evaluated. Without redefining the current notion of airtime, which neglects some overheads, a solution for fairness yields unfair results in practice. To our best knowledge, there is no study on airtime that has concerned indirect overheads, such as TCP ACK. Although we address a fundamental problem, our approach is practical. Instead of directly handling responsible airtime, a simpler fairness evaluation via the throughput fair share is introduced. For a simple and portable scheduling implementation, our schedule uses an estimator based on time measurement for responsible airtime of a data frame with no analysis of physical layer components. Our implementation is limited to download traffic in infrastructure-mode networks.

## 3. Necessity of a New Notion of Responsible Airtime

In order to validate our argument that the existing paradigms of airtime fairness yield false time-based fairness, we first analyze how time is actually occupied in two different data rate transmissions. The analysis shows that there exists a huge portion of time other than data transmission, and the amount highly varies depending on the data rate. Considering airtime only for fairness is irresponsible, and hence a new notion of airtime is needed for true time-based fairness.

### 3.1. Analysis of Actual Airtime in Transmission

Questions on airtime fairness come from the experimental observations shown in Table 1. Two simple cases were evaluated in the ns3 simulator, and the airtime of each case was analyzed. In both cases, there was a single node in the wireless LAN and TCP bulk traffic was downloaded. The only difference in the cases was the distance from the AP to the node and resulting data rate. Case 1 was 20 m and 54 Mbps, and Case 2 was 110 m and 6 Mbps, respectively. For each case, downloading was performed for 10 s, and the throughputs were different, as expected, because the higher data rate increased the number of frames transmitted for the experiment duration. An interesting point is that the sums of airtimes for each case are much different. 

As a general meaning of working time, their airtimes would be expected to be similar because both simulations were performed in the same saturated traffic for the same 10 s. However, as shown in the table, the 54 Mbps case is approximately 3.8 s less than the 6 Mbps case. It is also shown that in the 54 Mbps case, about 50% of the total operation time is used for purposes other than pure data transmission, whereas only 15% was used in the 6 Mbps case. When the airtime was extended to include per-frame overheads of DIFS, SIFS, and MAC ACK, which we call extended airtime in this study, the gap becomes less; however, there still exists a significant difference.

From the above observations, it can be understood why allocating equal airtime does not guarantee fairness in multiple data rates. Suppose that an AP schedules saturated download traffic between one 54 Mbps node and one 6 Mbps node. If the scheduler allocates the same amount of pure airtime, two nodes take different times for actual processing, where the former node requires more than 200% of the allocated pure airtime and the latter node requires approximately 120%. The actual result is uneven time sharing, where the slower node uses much less than the expected, i.e., the half of the link time. 

The observations have an alternate explanation as well. An example of perfect time-based fairness is the synchronous time-division multiple access (TDMA) with equal weight because it evenly divides the operation time among nodes. Suppose TDMA is applied in the above situation for 20 s. Then, each node can access the link in a dedicated manner for 10 s. Each of the two nodes would obtain the same throughput as in Table 1, and their airtimes would differ. To argue that allocating equal airtime is fair, one should argue that TDMA is not fair. 

This result is consistent with the public perception of airtime fair scheduling. Based on experience in airtime fair scheduling implementation at wireless routers, for example, Cisco ATF [17], the performance of a slow node becomes so worse than in FIFO that the connection is unstable. There may be some enhancements, including overheads; however, there are few implementations that are adequately recognized to be adopted in practice.

**Figure 1:**
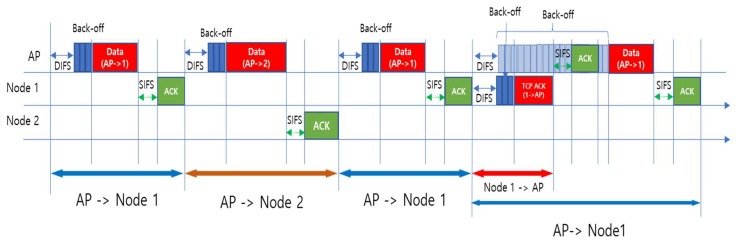
Typical Transmission Scenario.

It is clear that there exist more time-consuming components other than data frame transmissions. To determine what they are, the process of data transmission is analyzed in detail. Figure 1 shows a typical data download scenario, where the AP sends TCP data to Node 1 and Node 2 in the 802.11 DCF mode without RTS/CTS. A frame is transmitted through the sequence of DIFS → CW backoff → data frame → SIFS → MAC ACK. Although two transmitters are involved, the time for the whole sequence can be considered a single session and be charged as an extended transmission time of a data frame. The time for CW backoff is not easily predictable, especially when a frame collision is involved.

A complicated component is a TCP ACK message, which is a frame transmitted from Node 1 shown in the right half of the figure. First, it is disputable to whom its cost is to be charged. On one hand, it can be considered a part of the previous data transmission and then should be counted as indirect overhead. On the other hand, it might be counted independently of the data transmission from the AP. However, its number of occurrences is certainly related with the number of data transmissions from the AP. Second, its amount of time is not easily predictable. Its frequency of occurrence is variable since TCP has a complicated rule on sending an acknowledgement, which is affected by many factors. In addition, the cumulative semantics of a TCP acknowledgement message may be related with multiple data previously transmitted, and hence it is not easy to distribute the responsibility for its time cost. 

Another component is related with a frame collision. If a collision actually occurs, the time cost includes the wasted time, which extends to the longest of the collided frame transmission times. If a collision is avoided, there is no wasted time; nevertheless, it causes the backoff time to increase. The case is shown on the right side of Figure 1, where the AP backs off while Node 1 transmits a TCP ACK. Again, it is a question of to whom the cost should be charged. Certainly, the number of frame collisions for a node is also related with the number of data frames that are transmitted to a node and hence at which data rate the node operates.

In summary, a process of data transfer in a WiFi network contains other time-consuming components than the pure transmission time of the data frame transmission. The time costs of those other components are not easily determinable, which be a reason why airtime has been used in a vague manner. It is certain that a higher data rate yields more data, and more data causes higher overheads. Fair scheduling based on airtime without correctly counting those components, which is the way the current airtime fair scheduling operates, results in unfairness in reality.

### 3.2. Responsible Airtime

The above observation gives insight into what is allocated fairly among nodes for true time-based fairness: it is not pure airtime, rather, it is the total time cost. In other words, it is necessary to redefine the notion of airtime in airtime fair scheduling. There have been other similar notions of time, such as link occupation time [4]. Nevertheless, they still are limited for covering a time for collision resolution or an indirect time cost, such as a TCP ACK message from a receiver. 

The notion of responsible airtime is officially introduced here, as the total time cost covering all responsible time components. The correct wording might be “responsible time”; however, we choose “responsible airtime” because airtime is a more frequently recognized term in fair scheduling. A responsible airtime of a data transmission represents the total time cost of all components engaged in the data transmission. Since a component may span multiple actions, sometimes it is more useful in an aggregate manner. A responsible airtime of a node is also used to mean the total aggregate time cost for all data transmissions from and to the node throughout an operation or simulation. Time-based fairness in multi-rate networks is achieved when fairness in responsible airtimes of each node is achieved. 

A responsible airtime contains the time to use the link directly and indirectly, as well as all overheads and even some link idle time for back-off. Since it might have too many components to enumerate, it would be helpful to figure out its complement, i.e., what is not included in responsible airtime. There are only two components that are not related with the data transfer. One is overhead for wireless network management, such as beacon transmission, while the other is idle time due to the absence of traffic, which is different from idle time due to backoff. In a saturated traffic environment, which is assumed to occur by default for a fairness evaluation, the latter appears only at the initial transition interval to the steady state. Excluding those two aspects, any time interval should be a part of responsible airtime of a node. 

**Figure 2:**
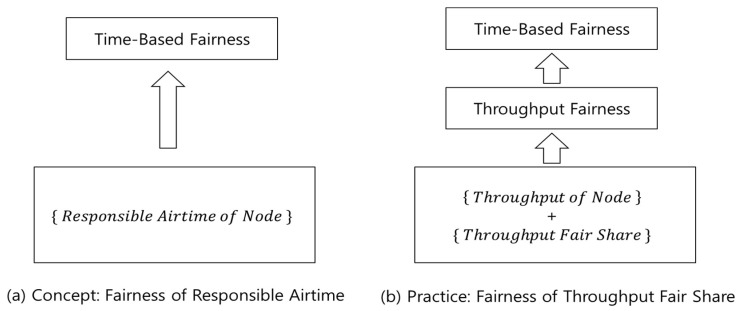
Responsible Airtime Fairness: Concept and Practice.

Conceptually, fairness based on responsible airtime can be addressed as usual. For example, the responsible times of each node are measured, and Jain’s Index [14] of them may be calculated to evaluate fairness. However, we believe that, unlike pure airtime, the actual responsible airtime of a node is difficult to measure or analyze from a scheduling result. The exact amount of responsible airtime of a frame, which is necessary for airtime fair scheduling, also seems to be not easy to calculate with its data length and data rate. Instead of making a complicated analysis, this study uses responsible airtime as a conceptual scale only. In practice, fairness is indirectly addressed via throughput, as shown in Figure 2.

First, we do not attempt to determine the responsible airtime of each data frame. An aggregate measure, i.e., the responsible airtime of a node, is concerned because it is simpler to handle and sufficient for long-term fairness. When the responsible airtime of a data frame is needed, such as in scheduling implementation, a statistical approximation is used. Second, the value of responsible airtime of a node is not directly determined either. Using the throughputs of the nodes, which are easily and accurately measurable, fairness is addressed. With the achieving ratio of the actual throughput to the fair share, this throughput fairness can be translated into fairness in responsible airtimes. This is the main topic of the next section.

## 4. Evaluation of Responsible Airtime Fairness

### 4.1. Throughput Fair Share

Assuming *N* nodes competing for *T* sec of responsible time under perfect time-based fairness, each node uses the same amount of responsible time, i.e., *T/N*, regardless of the data rate. We call this responsible airtime fair share. In principle, this responsible airtime fair share is the basis in deciding fairness; fairness basically means how closely each node achieves this fair share. However, the actual responsible airtime of a node is difficult to determine. 

To bypass this difficulty, we introduce the throughput fair share of a node, which is throughput that the node obtains for the responsible airtime fair share. Conceptually, it is a fair share in throughput when a node competes under the perfect time-based fairness. In a multi-rate wireless network, the responsible airtime fair share is the same for all the nodes. However, the throughput fair shares of each node may be different depending on the data rate. Similar to the responsible airtime fair share, throughput fair share can be used as a basis in deciding fairness. Fairness is represented by a metric of how close the actual throughput of each node is to its fair share. In fact, throughput fair share is the ideal throughput mentioned in the baseline property [3]. In multi-rate wireless networks with time-based fairness, a node should obtain a consistent level of throughput regardless of what rates at which the neighbor nodes operate, and the level is the one achieved when all neighbor nodes operate in the same data rate of the node. 

Overheads act as interference in achieving throughput. Throughput fair share is the throughput in allocating a sufficient airtime when all direct and indirect overheads are taken into account. Since fairness is evaluated using the achieving ratio of actual throughput to the throughput fair share, our fairness evaluation does not determine the exact amount of interference. However, the responsible airtime of a node takes account of overheads including TCP ACK.

Now we address our reasoning that fairness in responsible airtimes is handled via the fairness in throughputs. Let $rat_{i}$ be the responsible airtime of node i and {$rat_{i}$}, the set of $rat_{i}$, be the responsible airtimes of the nodes in a wireless network. Additionally, let $s^{rat}$ be the responsible airtime fair share, which is equal for all nodes. Both a responsible airtime fair share and an actual responsible airtime are dependent on how many nodes compete and the time length in which they compete. Hence, the correct notation is $s^{rat}\left(N,T\right)$ and $rat_{i}\left(N,T\right)$, where N and T are the number of nodes in the network and the competing time, respectively. Fairness studies usually compare scheduling results in a given environment. We use the simpler terms, $s^{rat}$ and $rat_{i}$, unless the environment is needed to be specified with N and T.

When a general fairness function is notated as Fairness(), for example, Jain’s Index function, the time-based fairness is described as $Fairness\left(\left\{rat_{i}\right\}\right)$. This is our conceptual approach, as illustrated in Figure 2a. Since $s^{rat}$ is constant for each node, without loss of generality:

(1) $$\;Fairness\left(\left\{rat_{i}\right\}\right)=Fairness\left(\left\{\frac{rat_{i}}{s^{rat}}\right\}\right)$$

Next, let $x_{i}$ be the actual throughput that node i obtains, and let $s_{i}^{x}$ be the throughput fair share of node i. Since the competing time is meaningless in the throughput, the correct notions are $x_{i}\left(N\right)$ and $s_{i}^{x}\left(N\right)$, respectively, where N is the number of nodes in the network. Again, $x_{i}$ and $s_{i}^{x}$ are used unless there is the need to distinguish the network environment. From the definition of throughput:

(2) $$\;\frac{size\;of\;data\;actually\;transmitted\;to\;node\;i}{expected\;size\;of\;data\;from\;fair\;share\;of\;node\;i\;}=\frac{x_{i}\times T}{s_{i}^{x}\times T}=\frac{x_{i}}{s_{i}^{x}}$$

We call this ratio $\frac{x_{i}}{s_{i}^{x}}$ the throughput achieving ratio of node i, denoted as $a_{i}$, which indicates how closely the throughput fair share is achieved at node i. If $a_{i}<1$, it means that, under fair achievement in that node, i achieves less throughput than expected. For a node, the amount of data transmitted is linearly proportional to its responsible airtime; thus:

(3) $$\;\frac{size\;of\;data\;actually\;transmitted\;to\;node\;i}{expected\;size\;of\;data\;from\;fair\;share\;of\;node\;i\;}=\frac{rat_{i}}{s^{rat}}$$

From Equations (2) and (3), we have:

(4) $$\frac{rat_{i}}{s^{rat}}=\;\frac{x_{i}}{s_{i}^{x}}\;=a_{i}$$

Finally, from Equations (1) and (4), we have:

(5) $$Fairness\left(\left\{rat_{i}\right\}\right)=Fairness\left(\left\{\frac{x_{i}}{s_{i}^{x}}\right\}\right)=Fairness\left(\left\{a_{i}\right\}\right)$$

Equation (5) says that the fairness of responsible airtimes can be determined via the fairness of throughputs, or, more precisely, the fairness of the throughput achieving ratios. This is our practical approach, as illustrated in Figure 2b.

The actual throughput of node i, $x_{i}$ is easily determined by measurement. The throughput fair share of node i, $s_{i}^{x}$, can be also measured; however, a separate experimental setup is needed. Since 802.11 MAC provides a fair chance to access the link for every node, all nodes can obtain the same throughput if their channel and traffic conditions are the same. When the number of competing nodes is N, we build an extra environment wherein there are N nodes whose channel conditions and application traffic are the same as those of node i. Every node consumes an equal amount of responsible airtime and obtains an equal throughput. $s_{i}^{x}$, in more precise notion, $s_{i}^{x}\left(N\right)$, is determined as the average of their throughputs. It is worthwhile to note that this measurement setup is the same as the basis environment in the baseline property. The throughput in the basic environment is equal to the throughput in the perfect airtime fairness, which is our throughput fair share. Our throughput achieving ratio, in another interpretation, indicates how closely the baseline property is maintained in effect.

One might doubt that the cost determining the throughput fair share is too costly. First, it might not be as expensive as the alternative of determining an actual responsible airtime. As explained in the previous section, considerable complexity can be expected, even if a fully detailed trace of network activity is available. Second, determining a fair share is needed per the network configuration. Unless the network configuration is changed, the same fair share is reused on all experiment results. The cost issue will be further explained with the experiment results in Section 6.

### 4.2. Proposed Time-Based Fairness Index

Many fairness indices have been proposed. Most of them focus on describing equity among competitors in a performance metric. For example, Jain’s Index [21] of throughputs at each node has been used as a representative indicator for individual fairness in a wireless network. Conceptually, equity in actual responsible airtimes among nodes could also be a good index for time-based fairness; equity in throughput-achieving ratios could be in our practical approach. However, there could be a better approach to represent time-based fairness than equity only because the fair share of each node is already determined.

As mentioned above, our fairness evaluation is based on the value of the throughput-achieving ratio at each node. If it is greater than one, there is a surplus, and if it is less than one, there is a shortage from the fair share. In time-based fairness, a node relinquishes equal performance to pursue system fairness. Hence, preserving each fair share could be critical for fairness. That is, the degree of shortage is significant while the surplus is not. Equity does not distinguish surplus and shortage. For example, Jain’s Index of {1.10, 1.10, 1.10, 0.70} is the same as that of {1.30, 0.90, 0.90, 0.90}; however, from the perspective of a shortage, they cannot be the same. This study adds a notion of deficiency to equity to explicitly describe how much shortage from the throughput fair share occurs at each node.

There are two factors to describe the degree of deficiency: the number of nodes suffering the shortage and the amount of shortage. In the spirit of max-min fairness, we confer more weight to the amount of the shortage. Generally, if one is treated unfairly, the whole is perceived to be unfair; however, the unfairness does not increase twice if the number of unfairness doubles. In the above example, one generally believes that {1.10, 1.10, 1.10, 0.70} is more unfair than {1.30, 0.90, 0.90, 0.90} because the former suffers up to a 30% shortage, whereas the latter suffers an only 10% shortage. We define the deficiency index, DI, as follows:

(6) $$DI=\max\left\{\max\left(1-a_{i}\;,\;0\right)\right\}$$

Since $a_{i}\geq 0,\;\max\left(1-a_{i}\;,\;0\right)$ has a positive value if $a_{i}<1$, and it indicates the relative shortage from the throughput fair share of node i. If $a_{i}\geq 1$, it is zero, which means that the surplus is simply ignored. DI is determined as the worst case, i.e., the largest shortage among the nodes. 

For the equity index, EI, the well-known Jain’s Index is used. Finally, the fairness index is defined as the product of the equity index and the complement of the deficiency index as follows:

(7) $$\begin{array}{ll} FI & =EI\times \left(1-DI\right)\\ & =\frac{{\left(\sum_{1}^{N}a_{i}\right)}^{2}}{N\sum_{1}^{N}a_{i}{}^{2}}\times (1-\max\;\left\{\max\left(1-a_{i}\;,\;0\right)\right\}) \end{array}$$

where *N* is the number of nodes. Since both DI and EI are between zero and one, FI is also between zero and one. Similar to Jain’s Index, the closer it is to one, the fairer it is. Table 2 shows fairness index values for some example cases of scheduling results.

## 5. Implementation of Responsible Airtime Fair Scheduling

### 5.1. Estimating the Responsible Airtime of a Frame

Now we check if there can be a scheduler that actually provides responsible airtime fairness. For a fast feasibility test, we limit our development to download traffic and choose simplicity over completeness. That is, the implementation goal is a simple scheduler at the AP that approximately performs responsible airtime fair scheduling. Assuming download traffic only, it is only half of the solution for fair scheduling. However, it is believed that checking the validity and applicability of the responsible airtime approach is an important first step. 

An essential component in scheduling is the responsible airtime of a frame to transmit. In order to determine it, we must count not only the frame length and data rate, but also the overhead, as mentioned above. Since long-term fairness is the issue, the exact responsible airtime for each frame is not necessary. We instead maintain an estimate, as in the following explanation, and apply it for the scheduler. 

The scheduler in the AP continues maintaining an expected responsible airtime of a frame for each node. Whenever a frame is transmitted to a node, its total time duration is measured as a sample time, and the expected value of the node is updated with the sample. The starting point of the duration is when the AP requests MAC access immediately after a frame is scheduled to transmit. The ending point is when the AP successfully receives the MAC ACK. If no frame intervenes, this duration covers the extended airtime explained in Section 3.1. An upward frame containing TCP ACK may intervene in the back-off interval, as shown in Figure 1. In this case, the duration also includes the transmission time of the TCP ACK. This case is considered not to be a problem and the time sample is valid because TCP ACK is also a part of the responsible airtime as indirect overhead. The source node of the TCP ACK frame is not distinguished owing to a rationale explained later. When a frame collision occurs in the duration, the time sample may be much greater than that of there being no collision. We simply discard this big sample; specifically, a sample greater than twice of the current expected is discarded in the test implementation, due to the following reason.

Ideally, the time cost for a TCP ACK should be included in the responsible airtime of the data frame that causes the TCP ACK. However, this correctness per data frame is not necessary for long-term fair scheduling. What is important in long-term performance analysis is whether its cost is counted toward the correct corresponding expected value. For a given a TCP ACK, the probability that it is caused by data frames to a certain node, say node *i*, is proportional to the occurrence frequency of data frames to node *i*. This is because the frequency of sending a TCP ACK is proportional to that of receiving a data frame. In our measurement rule where a TCP ACK is simply included in the outstanding data frame at the AP, the probability that a TCP ACK hits on a data frame to node *i* is also proportional to its occurrence frequency. Therefore, an expected responsible airtime based on our measurement is valid with respect to the TCP ACK overhead. In short, direct causality does not matter as long as overhead distribution is statistically correct. The rationale for this condition is similar to that of RED in congestion control at a router [22]. 

The overhead of frame collision is dependent on the number of collisions and the cost per collision. The former is certainly related with the number of data transmissions, and thus it may be handled in a similar way to the case of TCP ACK. However, unlike TCP ACK, the latter is widely varied depending on which nodes get involved. Integrating the cost of a collision without deterministic analysis may distort an estimation value. A lower data rate node sends less data and causes less collisions; however, the wasted time per collision is bigger because the transmission time of data frame is longer. From the experimental measurements, it is found that the overall collision overheads for each node might not be much different, regardless of their data rate. In precise, a lower data rate node causes slightly higher overhead than a higher data rate node when a frame length is longer 1000 bytes. This means that ignoring collision overhead may not affect fairness much, and at least not be harmful for a lower data rate. This is the reason why a big time sample, in which a collision is usually involved, is discarded. 

Certainly there exist traffic environments where these reasonings are not valid. One example is shown in Section 6. Our argument here is that how much accurately implementing airtime fairness can be an engineering choice though absolute correctness in deciding fairness is necessary. Our approximation is not perfect—however, it is effective—in most cases.

Our estimation method is a simple averaging of sample measurements. We tested many other estimators, such as the median or the minimum of time samples; however, the exponential moving average with a 0.1 smoothing factor was found to be a good fit through experiments. It is simple but adequate to cover variable cost components. This will be shown later in the experiments.

Some may doubt that the frame length and data rate are not considered in the expected responsible airtime of a frame. It is true that they are not explicitly and deterministically counted; however, it is also true that they are reflected in a time sample for the duration. It is certain that the frame length and data rate can be dynamically varied in real environments; nevertheless, they can eventually be covered by a moving average, albeit not instantaneously. Again, our implementation focus is a simple approximation of long-term time-based fairness. 

Meanwhile, our implementation based on time measurement can be highly portable. We do not directly handle any physical details, such as the data rate; we handle time duration as a synthetic output that integrates the details in the system. As a result, our implementation scheme is independent of the physical layer and hence is implementable purely in the queue management. As a result, it is applicable to any 802.11 physical standards or data rate adaptation mechanisms.

### 5.2. Responsible Airtime Scheduling

Most implementations of airtime fair scheduling are based on the deficit round-robin (DRR) [23], where the deficit is in the unit of bits, and the deficit counter of each node is reduced by its own quantum value, which is basically the weight of each node in weighted fair queuing [24]. Specifically, in airtime fair scheduling, the quantum values are based on the data rates of each node. A node with a higher data rate has a larger quantum value than a node with a lower data rate and is thus scheduled more frequently. If the ratios of the data rate are maintained in the ratios of the quantum values, the result is the same as the pure airtime fair scheduling. In order to cover the extended airtime or responsible airtime, a rule is needed that appropriately converts the data rate into the corresponding quantum value. However, this requires a complicated analysis of overhead.

Our scheduling algorithm is also based on DRR. Nonetheless, it is different in that it does not use quantum characteristics. The deficit counter is in the unit of time, that is, seconds. Whenever a frame to a node is scheduled to transmit, the counter of the node is set as its expected responsible airtime. That is, each deficit counter value indicates the time for which the corresponding node has used the link previously and hence must wait. All deficit counters are reduced at an equal pace, and only a node with a zero deficit is clear to be scheduled. The quantum approach is unnecessary because the expected responsible airtime already includes not only the data rate but also overhead. The long-term ratio of the times for which each node uses the link is the same as that of each expected responsible airtime. Thus, responsible airtime fairness can be achieved as long as the expected values are accurate.

**Figure 3:**
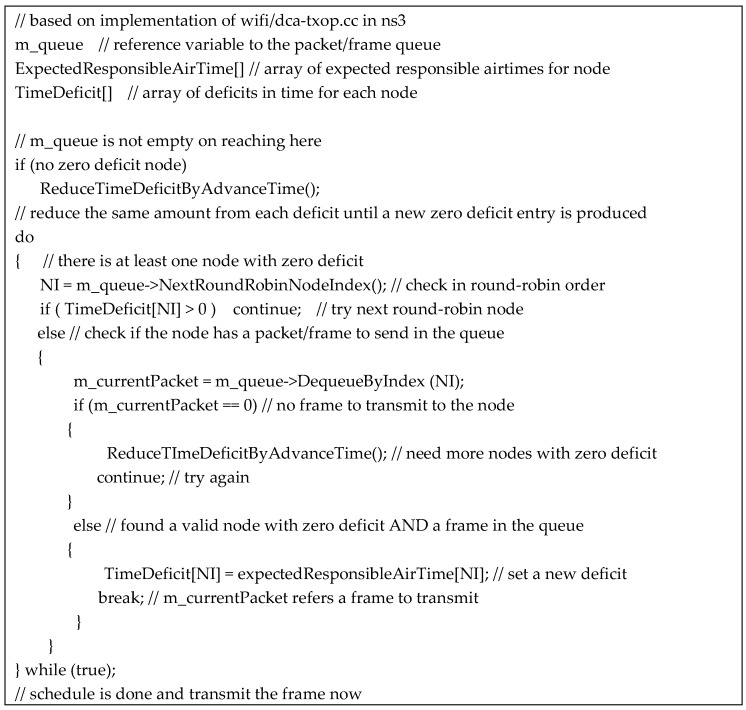
Code Segment of Responsible Airtime Fair Scheduling.

Figure 3 shows our scheduling algorithm in detail. The code is based on the implementation of the ns3 [25] wifi module; it is inserted into the scheduler part in dca-txop.cc. As the ns3 variable, m_queue refers the queue of frames to transmit, and m_currentPacket refers to the frame to transmit to next; our code must set m_currentPacket to the next frame in responsible airtime fair order. ExpectedResponsibleAirTime records the expected responsible time of each node, which is calculated from the measured time samples, as explained in the previous subsection. TimeDeficit is the key in scheduling and maintains a deficit value of each node in a nano-second scale.

The scheduler first equally reduces all entries of TimeDeficit until at least one entry is zero. Then, it checks each node in round-robin order if it is a valid node, which has a zero deficit and has a frame in the queue. If the node has a positive deficit, the next round is simply made because other nodes having a zero deficit are waiting. If the node has a zero deficit but no frame in the queue, reducing the deficits again is required for the work-conserving property before attempting the next round.

Applying the ns3 implementation to real APs will not be a major burden. For example, in openwrt, an open source project for wireless routers, modification at the following code portions are expected: adding some code to approximate a responsible airtime for each node can be achieved by measuring times (ath9k_hw_set_txq_props() in ath9k/mac.c and ieee80211_frame_acked() in mac80211/status.c) and modifying or adding packet scheduling code (ath_txq_schedule() in ath9k/xmit.c). The implementation will not impose additional overhead because it does not require any information exchange between an AP and nodes and the processing cost would not be significant. All operations are done at a relatively high level using system time and queue management. Therefore, this implementation is applicable to any 802.11 physical standards or data rate adaptation mechanisms. 

In order to construct a general responsible airtime scheduler, two modules should be implemented: one for determining the responsible airtimes for each node, and the other for implementing a distributed scheduler based upon the values of the responsible airtimes. Our approach to the second module will be to simply borrow the best existing solution, as the scheduler in the ns3 implementation borrowed the idea of the deficit round-robin. The two modules are independent of each other, and the responsible airtime approach is more related to the first module.

One principle in implementing the first module is that responsible airtime should be approximated without complicated analysis. This aim is difficult to achieve at a node locally because any measured duration includes a traffic queuing delay, which is not the case at the AP. In general, information exchange among nodes through the AP is necessary. The key problem is what kind of information is exchanged, and how this information is utilized in calculating responsible airtime. The amount of the overhead will be similar to those of other implementations, which is basically the cost required to implement distributed fair queuing. The other principle is to maintain compatibility with wifi standards, which is the most important in applying to the real devices.

## 6. Experiment Results

### 6.1. Validation of Fairness in TCP Traffic in a Basic Topology

**Figure 4:**
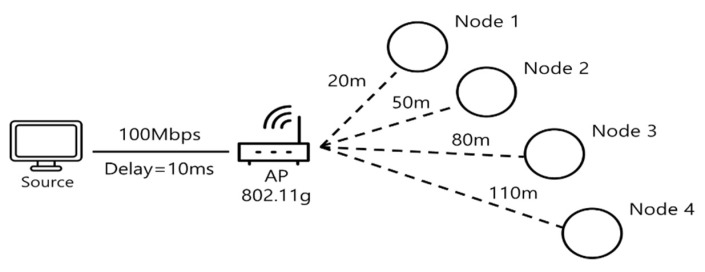
Basic Network Topology in Experiments.

The goal of the experiments was to compare the time-based fairness of scheduling methods, and all experiments were performed with the well-recognized simulation tool, ns3 [25]. As an example of multi-rate wireless networks, the experimental environment was set up as shown in Figure 4. Saturated TCP traffics were downloaded from the server in the wired side to each of the wireless nodes through the 802.11g AP, where the wireless nodes were separated with a distance sufficient to operate in different data rates. With no specific reference, all performance data were obtained from this setup.

First, the throughput fair shares of each node are determined. To this end, for each node, we set up a separate environment wherein the basic topology is changed so that all nodes are at the same distance to the AP. For example, for the throughput fair share of Node 1, the environment consists of four nodes of which the distances are the same 20 m as Node 1. Since it is a situation where four instances of Node 1 are competing, the average of throughputs at each node can be used as the throughput fair share of Node 1. Table 3 shows the throughput fair shares of each node in the basic topology, where the traffic type is a TCP bulk transfer. The table also shows each node’s data rate and aggregate pure airtime for the throughput fair share, just for the contrast. The throughput fair shares are not directly proportional to the data rates, which can be explained as being due to overheads. The aggregate pure airtimes are obviously not equal. To be fair, with counting all overheads the faster node (Node 1) should use less pure airtime than the slower node (Node 4).

Making a real environment setup to evaluate the throughput fair share of a node may be more difficult than making it in simulation. Specifically, placing wireless nodes side by side may not guarantee physically identical channel conditions. However, we believe that it is feasible to build a setup that gives a consistent average in throughput among nodes within the experimental error range.

We evaluated the responsible airtime fairness of four scheduling schemes, as shown in Table 4. FIFO is the default scheduler of a current router that suffers a performance anomaly. It results in poor throughput achieving ratios at faster nodes, and the fairness index is approximately 0.34. ARF is the pure airtime fair scheduler, which ignores overhead effects. It causes slower node underachieving, and the fairness index is limited to approximately 0.73. RAF is our responsible airtime fair scheduler. It shows slight underachieving in faster nodes, although the fairness index is approximately 0.97. QDRR is a deficit round-robin scheduler whose quantum value is manually adjusted to fit the throughput fair shares. It is the best scheduler that is intended for the fairness upper-bound. In summary, our simple implementation provides reasonable responsible airtime fairness for TCP traffic, which indicates that the average of measured time durations for frame transmission approximates well with the responsible airtime of a frame.

**Figure 5:**
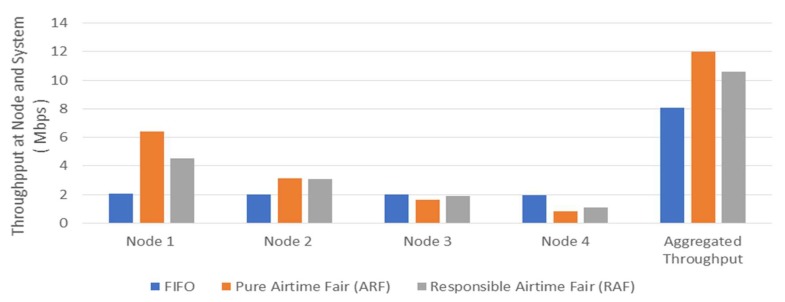
Throughput Performance in Fair Scheduling.

Achieving fairness certainly influences system performance. As shown in Figure 5, FIFO scheduling pursuits fairness in throughput of each individual node, and hence the aggregated throughput is the lowest because it requires sacrifice of a higher data-rate node in airtime. Pure airtime fair scheduling pursues fairness in airtime among nodes, and it gives the highest aggregated throughput because a higher data rate node has more chance to use the link. However, pure airtime fair scheduling without counting all overheads correctly is biased in favor of a higher data rate node. More data transmissions require more overhead and thus neglecting overheads is more beneficial for a higher data rate node which transmits more data during the same airtime. Responsible airtime fairness scheduling pursues fairness in total time usage with counting all responsible overheads. It removes the bias for a higher data rate node and yields a better performance in a lower data rate node. However, the aggregated throughput is lower than pure airtime fair scheduling.

### 6.2. Fairness in Various Traffic and Network Environments

We investigated the responsible airtime fairness varying traffic and network attributes. First, we tested how the responsible airtime fairness responds in UDP download traffic. Table 5 shows the results, along with the throughput fair share in the UDP of each node. Since UDP does not add extra overhead, their throughput fair shares are higher than those of TCP, and they are more closely related with their data rates. For the same reason, pure airtime (ARF) yields better results than TCP, as does our RAF, whose fairness index is almost 1.0. Again, QDRR was assessed just for comparison. QDRR of UDP has quantum values that differ from those of QDRR of TCP because the effects of the data rate to responsible airtime in the two cases are different.

Next, an experiment was conducted in an environment where TCP download and UDP download traffic were mixed. Specifically, Nodes 1 and 3 received TCP traffic and Nodes 2 and 4 received UDP traffic. The throughput fair shares of Node 1 and 3 were the same as those of the TCP experiment, respectively, and those of Nodes 2 and 4 were the same as UDP. As shown in Table 6, the fairness index of RAF is only 0.88, and two UDP nodes suffer deficiency. This can be explained as follows. In our simple implementation, the time cost for a TCP ACK was simply included into the duration of the outstanding frame at the AP without distinguishing its source node. In a homogeneous traffic environment, this causes no problem because the cost distribution based on the occurrence frequency is consistent to real causality. However, in a heterogeneous traffic environment, where TCP traffic causes TCP ACKs, whereas UDP traffic does not, the simple rule is effective. Specifically, a TCP ACK frame may include a time sample of a UDP data frame, which causes the estimation of the responsible airtime of a UDP traffic node to be longer and that of a TCP node to be shorter. As a result, UDP nodes underachieve and responsible airtime fairness is degraded as much as the overhead is miscalculated.

Regarding other scheduling algorithms, FIFO causes an extreme unbalance between TCP nodes and UDP nodes because it cannot isolate non-congestion-controlled UDP traffic. In ARF, where overhead is not counted at all, TCP nodes obtain higher achieving ratios than in the homogenous traffic because the effect of offsetting overhead is higher in TCP traffic. In QDRR, we intentionally reused the quantum values in TCP and UDP homogenous traffic, and the fairness index was 0.95. This means that the new proportional quantum values were required for a new traffic environment. This experiment demonstrated the limits of our simple implementation as well as the inherent complexity in determining correct responsible airtimes.

In the next experiment, mobility was added to the basic topology. Nodes 1 and Node 4 moved to each other’s position through the experiment time, while Nodes 2 and Node 3 remained at their respective positions. Table 7 shows the results. The throughput shares of Nodes 1 and Node 4 are newly determined. The purpose of this experiment was to check the adaptability in varying data rates. RAF shows almost the same fairness index as the case of no mobility shown in Table 4. FIFO also provides a better fairness index, although it is not because it has adaptability. It is because the channel conditions become more equitable than the case of no mobility. QDRR with the quantum value of the previous TCP experiment yields a much poorer fairness index because the quantum is fixed while the data rate is being changed. This result indicates that a dynamic adjustable quantum value is necessary in a wireless network environment. ARF is found to have some adaptability because the pure airtime of a data frame reflects changes in the data rate, although it is far from fair.

Another purpose of this experiment was to validate the baseline property. In true time-based fairness, Nodes 2 and Node 3 should maintain their performances regardless of the data rates at which other nodes, i.e., Nodes 1 and Node 4, operate. In the experiment, Nodes 1 and Node 4 continued changing their data rates as they moved. In comparing the results of Table 4, only RAF produces almost the same performance. We conclude that RAF well satisfies the baseline property and hence the true time-based fairness.

Since our implementation does not explicitly account for frame length, we also tested how each scheduling reacts on variable frame length. To maximize the length variation, we made the data length of the UDP application random in a uniform distribution between 100 and 1024 bytes. As shown in Table 8, RAF shows an approximate 0.98 fairness index, which is only 0.1 lower than the fixed length case in Table 5. This means that the exponential averaging well covers the effect of variation in the frame length on responsible airtime. Other scheduling methods show similar fairness pattern to the experiment of mobility shown in Table 7.

Finally, to validate the independence of the physical layer and portability of our implementation, we tested our responsible airtime fair scheduling on 802.11ac networks. 802.11ac has different physical characteristics from 802.11g, for example, it has a 5 GHz band and MIMO streams. In principle, determining the responsible airtime in 802.11ac requires a new analysis because new physical factors should be considered. Our implementation abstracts all physical details into the time duration. Therefore, it can be ported in 802.11ac as it currently exists. For a fair assessment, it should be checked whether those physical factors are correctly abstracted. Table 9 shows the results in 802.11ac, and our RAF gives a reasonable fairness index value again. Therefore, it could be said that the responsible airtime is predictable without analyzing the details of the physical layer. We did not test QDRR in this example because it was obvious that the quantum values in 802.11g have no meaning in 802.11ac.

## 7. Conclusions

For true time-based fairness, this paper introduced the responsible airtime concept, which includes not only the data transmission time, but also all overheads, even including a TCP ACK segment in TCP traffic. Responsible airtime is conceptually clear but difficult to directly handle. We thus developed a method to evaluate time-based fairness via throughput measurement. How closely the baseline property is satisfied is used as the barometer for fairness, and the fairness index is also extended to reflect a deficiency. A simple scheduling method at the AP was also developed, and the feasibility of responsible airtime fairness was validated through experiments in various traffic and network environments.

We believe that this study is unique in that fairness itself in airtime fairness was addressed. No study has addressed quantitative fairness with consideration of overheads such as TCP ACK. The responsible airtime approach is novel in many aspects, ranging from the concept to the evaluation method and implementation. Our solution for time-based fairness is highly practical and is thus applicable in real-world routers.

There may be at least two directions for further research on this topic. The first is to make the implementation more precise. One obvious point to be improved is to count the overhead of collisions deterministically in the responsible airtime of the data frame that causes it. The second direction is to apply our responsible airtime approach to upload traffic in wireless networks. Without QoS support at a MAC level, such as 802.11e, a new implementation scheme is required because centralized control is not possible. Some techniques in distributed packet scheduling, such as distributed fair queuing [26] and proportional fair allocation [2] could serve as a foundation; however, expecting responsible airtime would be necessary.

## Figures and Tables

**Table 1:** Airtime Analysis (TCP Bulk Download Traffic, 802.11g Simulation for 10 s).

Parameter	CASE 1 20 m, 54 Mbps Single Node	CASE 2 110 m, 6 Mbps Single Node
Throughput	18.657 Mbps	4.277 Mbps
Number of Data Frames Transmitted (1084 Bytes/Frame)	22,775	5221
Sum of (Pure) Airtimes for Data Frame Transmission	4.654 s	8.426 s
Sum of Extended Airtimes Including Overheads (DIFS, SIFS, MAC ACK)	7.493 s	8.693 s

**Table 2:** Fairness Index of Sample Cases.

Case (Set of Throughput Achieving Ratio)	Equity Index	Deficiency Index	Fairness Index
{1.0, 1.0, 1.0, 1.0}	1.00	0.00	1.00
{0.9, 0.9, 0.9, 0.9}	1.00	0.10	0.90
{1.3, 0.9, 0.9, 0.9}	0.97	0.10	0.87
{1.1, 1.1, 1.1, 0.7}	0.97	0.30	0.68

**Table 3:** Throughput Fair Share for TCP Traffic in the Basic Topology (4 nodes, 10 s).

Parameter	Node 1	Node 2	Node 3	Node 4
Distance from AP (m)	20	50	80	110
Data Rate (Mbps) (Rate Adaptation: IDEAL)	54	24	12	6
Throughput Fair Share (Mbps)	4.681483	3.109867	1.906934	1.059533
Measured Aggregate Pure Airtime (s)	1.10585	1.512145	1.791905	1.9411

**Table 4:** Throughput Achieving Ratio and Fairness Index in TCP Traffic.

Scheduling Algorithm	Node 1	Node 2	Node 3	Node 4	Fairness Index
FIFO	0.444118	0.646696	1.054646	1.842467	0.344713
Pure Airtime Fair (ARF)	1.371370	1.004525	0.848570	0.768302	0.729057
Responsible Airtime Fair (RAF)	0.965460	0.998572	1.000086	1.023215	0.965048
Quantum Deficit Round-Robin (QDRR) (Manually Adjusted Quantum)	0.994558	0.98893	0.986812	1.007832	0.986745

**Table 5:** Throughput Achieving Ratio and Fairness Index in UDP Traffic.

Scheduling Algorithm	Node 1	Node 2	Node 3	Node 4	Fairness Index
Throughput Fair Share (Mbps)	6.081147	3.801803	2.250381	1.221101	
FIFO	0.387026	0.619064	1.045850	1.927408	0.286828
Pure Airtime Fair (ARF)	1.289889	1.006858	0.881419	0.816449	0.790332
Responsible Airtime Fair (RAF)	0.997121	0.995934	0.993175	1.005365	0.993155
Quantum Deficit Round-Robin (QDRR) (Manually Adjusted Quantum)	0.998481	0.996364	0.993432	1.001612	0.993423

**Table 6:** Throughput Achievement Ratio and Fairness Index in TCP/UDP Mixed Traffic.

Scheduling Algorithm	Node 1 (TCP)	Node 2 (UDP)	Node 3 (TCP)	Node 4 (UDP)	Fairness Index
Throughput Fair Share (Mbps)	4.681483	3.801803	1.906934	1.221101	
FIFO	0.022497	2.020314	0.013938	1.638232	0.007031
Pure Airtime Fair (ARF)	1.453305	0.90483	0.897586	0.733932	0.683151
Responsible Airtime Fair (RAF)	1.008875	0.882959	1.054215	0.956795	0.87714
Quantum Deficit Round-Robin (QDRR) (TCP/UDP Quantum Reused)	1.047524	0.95133	1.037826	0.955606	0.949421

**Table 7:** Throughput Achieving Ratio and Fairness Index in TCP Traffic with Mobility.

Scheduling Algorithm	Node 1	Node 2	Node 3	Node 4	Fairness Index
Throughput Fair Share (Mbps)	2.434094	3.109867	1.906934	2.511789	
FIFO	0.867296	0.689808	1.107770	0.842424	0.670282
Pure Airtime Fair (ARF)	1.168780	1.044089	0.874818	1.156108	0.864145
Responsible Airtime Fair (RAF)	1.015715	0.972703	0.988916	0.987949	0.972466
Quantum Deficit Round-Robin (QDRR) (TCP Quantum in Table 4)	1.584530	0.819433	0.818794	0.352641	0.283379

**Table 8:** Throughput Achieving Ratio and Fairness Index in Variable Length UDP Traffic.

Scheduling Algorithm	Node 1	Node 2	Node 3	Node 4	Fairness Index
Throughput Fair Share (Mbps)	4.205086	2.920385	1.872179	1.074929	
FIFO	0.465375	0.667175	1.036752	1.820031	0.366719
Pure Airtime Fair (ARF)	1.064256	1.167587	0.943609	0.826082	0.812755
Responsible Airtime Fair (RAF)	0.977167	1.007989	1.003903	0.999893	0.977027
Quantum Deficit Round-Robin (QDRR) (UDP Quantum in Table 5)	1.071074	1.046437	0.96383	0.917568	0.914067

**Table 9:** Throughput Achieving Ratio and Fairness Index in 802.11ac TCP Traffic.

Scheduling Algorithm	Node 1 (20 m)	Node 2 (40 m)	Node 3 (60 m)	Node 4 (80 m)	Fairness Index
Throughput Fair Share (Mbps)	9.524336	9.14175	8.036446	6.65848	
FIFO	0.861723	0.896692	1.015551	1.229720	0.844278
Pure Airtime Fair (ARF)	1.315550	1.142832	0.867660	0.708023	0.671318
Responsible Airtime Fair (RAF)	1.008465	1.013723	0.998728	1.009420	0.998698
